# Supplementary material for: The causal relationship between the human gut microbiota and pyogenic arthritis: a Mendelian randomization study
Source: Front Cell Infect Microbiol. 2024 Nov 26;14:1452480. doi: 10.3389/fcimb.2024.1452480 (PMC11629706; doi:10.3389/fcimb.2024.1452480)
Supplement: Supplementary file 1 [file DataSheet1.zip › mendelian test/ebi-a-GCST90027576.csv_leave_one_out.pdf]

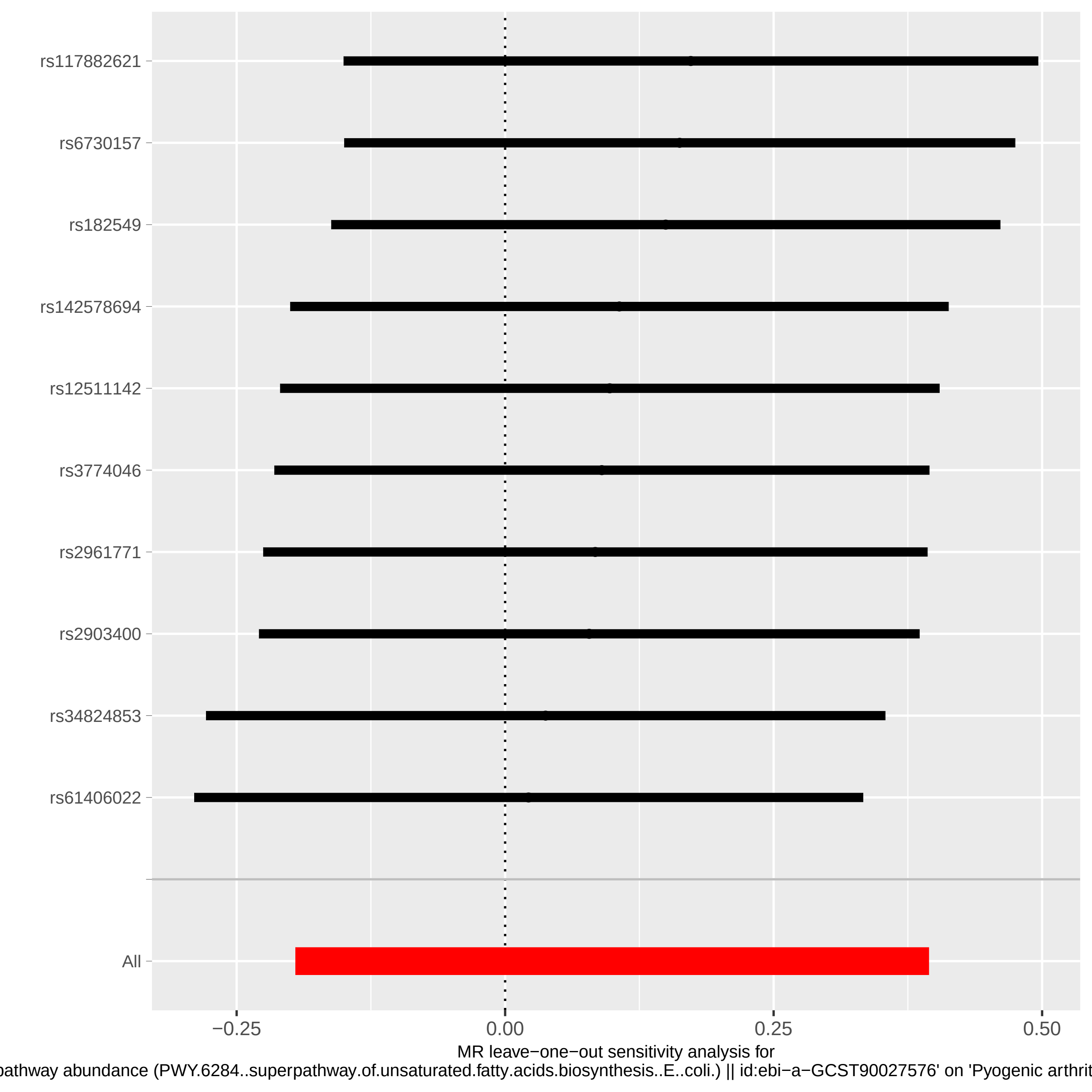

rs117882621
rs6730157
rs182549
rs142578694
rs12511142
rs3774046
rs2961771
rs2903400
rs34824853
rs61406022
All
−0.25
0.00
0.25
0.50
MR leave−one−out sensitivity analysis for
pathway abundance (PWY.6284..superpathway.of.unsaturated.fatty.acids.biosynthesis..E..coli.) || id:ebi−a−GCST90027576' on 'Pyogenic arthrit
